# Mapping the Salt Stress-Induced Changes in the Root miRNome in Pokkali Rice

**DOI:** 10.3390/biom10040498

**Published:** 2020-03-25

**Authors:** Kavita Goswami, Deepti Mittal, Budhayash Gautam, Sudhir K. Sopory, Neeti Sanan-Mishra

**Affiliations:** 1 Plant RNAi Biology Group, International Centre for Genetic Engineering and Biotechnology, Aruna Asaf Ali Marg, New Delhi 110067, Indiasopory@icgeb.res.in (S.K.S.); 2 Department of Computational Biology and Bioinformatics, Jacob School of Biotechnology and Bioengineering, Sam Higginbottom university of Agriculture, Technology and Sciences, Prayagraj (Formally Allahabad) 211007, India

**Keywords:** microRNA, expression profiles, salt tolerance, miR-eQTL, degradome, Pokkali root

## Abstract

A plant’s response to stress conditions is governed by intricately coordinated gene expression. The microRNAs (miRs) have emerged as relatively new players in the genetic network, regulating gene expression at the transcriptional and post-transcriptional level. In this study, we performed comprehensive profiling of miRs in roots of the naturally salt-tolerant Pokkali rice variety to understand their role in regulating plant physiology in the presence of salt. For comparisons, root miR profiles of the salt-sensitive rice variety Pusa Basmati were generated. It was seen that the expression levels of 65 miRs were similar for roots of Pokkali grown in the absence of salt (PKNR) and Pusa Basmati grown in the presence of salt (PBSR). The salt-induced dis-regulations in expression profiles of miRs showed controlled changes in the roots of Pokkali (PKSR) as compared to larger variations seen in the roots of Pusa Basmati. Target analysis of salt-deregulated miRs identified key transcription factors, ion-transporters, and signaling molecules that act to maintain cellular Ca^2+^ homeostasis and limit ROS production. These miR:mRNA nodes were mapped to the Quantitative trait loci (QTLs) to identify the correlated root traits for understanding their significance in plant physiology. The results obtained indicate that the adaptability of Pokkali to excess salt may be due to the genetic regulation of different cellular components by a variety of miRs.

## 1. Introduction

Unfavorable environmental changes exert abiotic stresses on plants, which hamper their development, negatively influence their life span, and cause yield losses [1]. Soil salt (mainly NaCl) is an important abiotic stress factor that creates osmotic imbalance, ion toxicity, and water deficiency in plants [2,3]. This affects the enzymatic and metabolic activities [4,5], leading to improper growth and nutritional deficiency [6]. It has been reported that under salinity, the plant height decreases significantly [7,8] and the lateral root initiation is distorted [9].

Plants have the ability to modulate their genetic machinery to overcome salinity stress. The components of stress response pathways involve signal transduction molecules, ion transporters, ROS scavengers, and cellular machinery for maintaining osmotic homeostasis. The glyoxylase (Gly) pathway has also been associated with the salt stress response in plants as it acts by removing the cellular toxicity and regulating glutathione (GSH) homeostasis [10]. Among the well-understood pathways are calcium-responsive SOS3-SOS2 protein kinases that activate the SOS1 ion transporters [11,12] and ankyrin-repeat motif-associated transmembrane proteins that influence the Abscisic Acid (ABA)-induced ROS accumulation [13]. The salt-tolerant rice cultivar Pokkali has been reported to contain a more active antioxidant defense system for efficient scavenging of the H_2_O_2_ as compared to salt-susceptible rice cultivars [14]. 

Changes in the soil salt concentration lead to dynamic responses in roots that influence the efficiency of water extraction and ion exclusion [15]. Induced salinity leads to changes in biochemical, molecular, and physiological interactions in the roots [16,17]. Variation in adaptability of a plant to changing soil conditions, to a great extent, relies upon appropriation of its root system architecture (RSA), which is essentially controlled by cell division and elongation in primary and lateral roots [18]. The RSA not only regulates the transport of water and minerals, but also communicates appropriate signals to the other parts of the plant. The proper elongation, distribution, and structure of roots within the soil are adversely affected by various stress conditions, such as salinity, water deficit, and nutrient deficiency [19,20]. Therefore, the root morphology, complexity, and differences between varieties leave much to be learned about the genetic regulation of plant responses.

The role of microRNAs (miRs) in the regulation of root growth has been studied in various plants, such as rice, maize, banana, chickpea, and Arabidopsis [3,17,21,22,23,24,25,26]. The miRs are small (20–24 nucleotides), noncoding, regulatory RNAs that are encoded by specific genes. They participate in numerous biological processes by regulating gene expression, mainly at the post-transcriptional level. Studies of Arabidopsis have reported that miR160 is essential for root tip growth and gravity sensing by regulating Auxin Response Factors, ARF10, ARF16, and ARF17 [27,28]. The lateral root growth is regulated by the action of miR393 on transport inhibitor response 1 (TIR1) and auxin signaling F-BOX 2 (AFB2). A role for miR390 through its targets ARF2, ARF3, and ARF4 has also been implicated [29,30]. miR408 and miR528 regulate root cap formation, lateral root development, and root elongation [23]. miR165/166 and miR390 also regulate primary root and lateral root development, respectively [25,30]. Likewise, miR167 regulates the activity of ARF6 and ARF8, which are positive regulators of adventitious root growth in soybean, Arabidopsis, and rice, while miR828 regulates root hair patterning [30].

In this study, we mapped the expression profiles of miRs in the roots of Pokkali (PK) when grown in the presence and absence of salt. To build these profiles, comparisons were made using the salt-sensitive cultivar Pusa Basmati (PB). This analysis identified crucial miR:mRNA interaction nodes that are genetically regulated in PK roots to minimize the effect of salt stress. These miR:miRNA nodes were mapped to the Quantitative trait loci (QTLs) to identify their correlated root traits for understanding their significance in the adaptation of PK under saline environments.

## 2. Materials and Methods

### 2.1. Plant Material

Mature seeds of Pokkali and Pusa Basmati rice were surface-sterilized with 10% commercial bleach for 5 min, washed thoroughly with sterile water, and placed on germinating sheets. The seeds were grown under controlled conditions of temperature (28 ± 2 °C), relative air humidity (70%), 16/8h light/dark cycle, and high light intensity (>700 micro mol PAR m^−2^ s^−1^). Salt treatment was provided using 200 mM NaCl for 15 days. For further analysis, root tissues were harvested from four sets of 15-day-old seedlings grown in the absence and presence of salt, viz. Pusa Basmati normal root (PBNR), Pokkali normal root (PKNR), Pusa Basmati salt-grown root (PBSR), and Pokkali salt-grown root (PKSR). Three biological replicates were used for each set.

### 2.2. Small RNA Library Construction and Sequencing

One gram of tissue was used for total RNA isolation using guanidine iso-thiocyanate (GITC) protocol, as described previously [31]. The small RNA (sRNA) fraction was enriched by LiCl precipitation [31] and the size range of 18-30nt was fractionated using 15% denaturing PAGE. These were used for library construction as per the manufacturer’s (Illumina, USA) protocol. The size, purity, and concentration of samples were checked at each step. Samples from three biological replicates were pooled for library construction and deep sequencing was performed on the Illumina HiSeq 2000 platform. 

**Figure 1:**
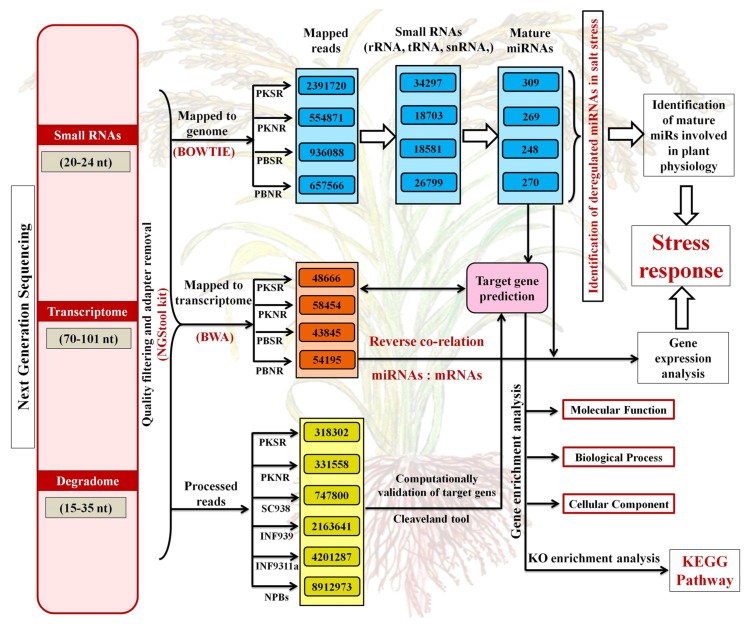
Overview of the analysis of sequencing data for identifying the microRNAs (miRs), mRNAs (transcripts), and cleaved target transcripts from the small RNA, transcriptome, and degradome libraries, respectively. Each panel provides information on the total number of mapped reads. The number of miRs predicted from each library is also indicated. The different arrows indicate the flow chart for performing the analysis. The samples include root tissues from Pokkali grown in the presence of salt (PKSR), Pokkali grown in the absence of salt (PKNR), Pusa Basmati grown in the presence of salt (PBSR), and Pusa Basmati grown in the absence of salt (PBNR). SC938, INF939, INF311a, and NPBs were degradome libraries obtained from open sources to validate the miR targets.

The reads were pre-processed to remove low-quality reads using the NGS tool-kit (http://www.nipgr.res.in/ngsqctoolkit.html) [32], followed by trimming out the adaptor sequence from the 3′ end. For rice miR identification, sRNA reads mapped to the genome were selected and aligned with the sequences downloaded from miRBase release 21 (http://www.mirbase.org/) using the Bowtie tool [33]. Only perfectly aligned sequences were considered as mature miRs (Figure 1). The accessions of the sequenced files are provided in Supplementary-File 1. 

### 2.3. mRNA and Degradome Sequencing and Analysis

A total of 1 ug total RNA from three biological replicates was used for transcriptome and degradome library preparation, as per established protocols [34], followed by sequencing on GAII (Illumina). Sequencing results were filtered to remove low-quality reads and the adapter sequence. Using burrow wheeler alignment software (version BWA-0.7.17, UK), these filtered reads were mapped to the *Oryza sativa* (rice), transcript, MSU Rice Genome Annotation (Figure 1). The transcript read length was in the range of 70-101 nt. 

Degradome sequencing was conducted to identify the transcripts cleaved by the miRs. The libraries were sequenced using the 5′ adapter only, resulting in sequencing of the first 36 nucleotides of the inserts that represented the 5′ ends of the mRNAs [34]. The results were further validated using Cleveland [35], with a specified *p*-value ≤ 0.05. Gene annotation (GO) enrichment analysis was performed for the target genes using AgriGO, in order to identify their biological functions [36].

### 2.4. Expression Analysis of miRs and Target mRNA 

The abundance of mature miR and mRNA sequences in specific libraries were determined and normalized as transcript per million (TPM) and Reads per Kilobase of transcript per Million (RPKM), respectively. For differential expression analysis, fold change was calculated as the ratio of normalized expression in the presence of salt to normalized expression in normal (absence of salt) conditions [34,37]. Log fold change was then calculated using the formula Log fold = log (N,2), where N represents the fold change. The negative and positive log2 value for miRs indicates their down regulation and up regulation in the presence of salt, respectively.

### 2.5. TaqMan PCR Assay

Total RNA was isolated using the miRNAeasy mini kit (Qiagen, USA) and quantified using Nano drop 1000 (Thermo scientific, USA). The TaqMan^®^ probes (Thermo scientific, USA) were custom designed for selected rice miRs. RT master mix was prepared using the TaqMan^®^ miR reverse transcription kit components. For the PCR reaction, 10 µL TaqMan^®^ 2× universal PCR master mix, 7.67 µL nuclease free water, 1 µL TaqMan^®^ small RNA assay (20×), and 1.33 µL RT reaction products were combined in a 96-well reaction plate. The reaction plate was briefly centrifuged and sealed with optical adhesive film. An experiment was created using the following thermal cycling conditions: 95 °C for 10 min for the activation of AmpliTaq Gold^®^ DNA Polymerase, followed by 40 cycles of amplification at 95 °C for 15 s for denaturation, and 60 °C for 60 s for annealing. The reaction plate was loaded on to the StepOnePlus™ real-time PCR instrument and results were analyzed using their Expression Suite software V1 (Thermofisher, Waltham, MA, USA). 

### 2.6. miR-eQTL Search and Mapping

miRs that expressed quantitative trait loci (miR-eQTLs) were predicted by designing a Perl script. For the prediction of miRs associated with QTLs, the precursor sequences were taken from the miRBase database. The number of traits associated with different physiological, anatomical, cellular, and biological categories was collected. The Mapchart tool [38] was used to assign and map the location of each QTL on the chromosome.

## 3. Results

### 3.1. Expression Profiling of miRs in Roots 

Small RNA and transcriptome profiles of Pokkali (PK) and Pusa Basmati (PB) root tissues grown in the presence (SR) and absence (NR) of 200 mM NaCl were generated by RNAseq analysis (Figure 1). This identified 269 (PKNR), 309 (PKSR), 270 (PBNR), and 248 (PBSR) miRs from each library (Supplementary-File 2). Overall, these included 155 miRs belonging to 23 conserved miR-families and 208 miRs aligning to 23 non-conserved miR-families. The ancient miRs present across different land plants and in the non-flowering moss represent the evolutionarily conserved miRs, while lineage-specific miRs that generally only exist in limited species are referred to as evolutionarily non-conserved. It was observed that in the presence of salt (stress), the total number of miRs increased by 34% in PK roots and decreased by 22% in PB roots (Supplementary-File 3).

**Figure 2:**
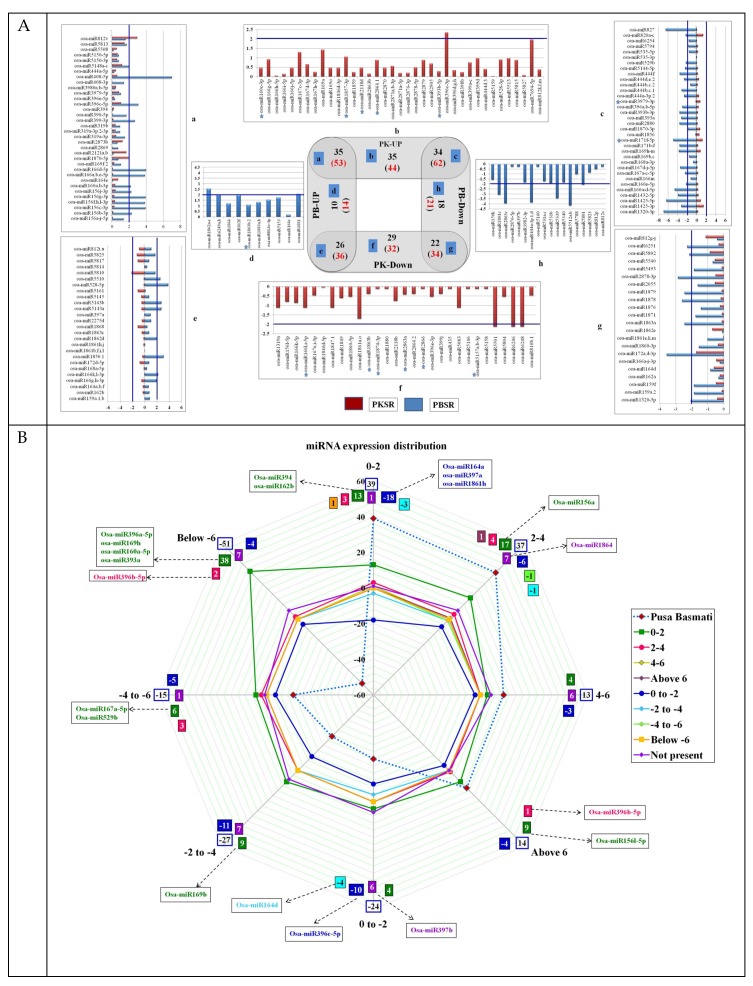
Expression profiles of osa-miRs. (**A**) Venn diagram representing the distribution and expression profiles of miRs across the four root libraries under control and experimental conditions. Graphs corresponding to panels a, c, e, and g represent the fold changes in miR expression in the presence of salt. Graphs corresponding to panels b, d, f, and h represent the digital expression of miRs. The samples include root tissues from Pokkali (PK) and Pusa Basmati (PB) grown in the presence (SR) and absence of salt (NR). (**B**) Graphical representation of fold change in miR expression levels in PB (dotted lines) and the relative changes observed in PK roots. A color code is used to indicate the fold range and number of miRs showing deregulation within that range.

The comparative analysis of all four libraries identified that 185 miRs belonging to 69 distinct families were expressed in all conditions (Supplementary-File 3). Among these, 53 miRs were up regulated and 34 miRs were down regulated in the presence of salt in both PK and PB roots (Figure 2(Aa,g)). Within this set, osa-miR408-5p, osa-miR156(b,c,g,f,h,j,l-3p and a-j-5p), osa-miR160(a,b-3p), osa-miR166(a,b,d,e-5p), osa-miR390-3p, osa-miR396c-5p, osa-miR1862a-c, osa-miR5813, and osa-miR6249(a,b) were highly up regulated in PBSR, while variation levels were lower in PKSR. Levels of osa-miR812v and osa-miR1870-5p were highly up regulated in PKSR (Figure 2(Aa)). Among the down regulated miRs, osa-miR172a,d-3p, osa-miR1879, osa-miR1863a, osa-miR1878, osa-miR2878-3p, osa-miR1861c, osa-miR5339, osa-miR444a-3p.1,d, osa-miR2863c, and osa-miR5512a,b showed higher variations in PBSR than PKSR (Figure 2(Ag)). 

In total, 62 miRs were down regulated in PBSR, but did not show significant changes in their levels in PKSR (Figure 2(Ac)) and among these, osa-miR827a-c, osa-miR171f-5p, osa-miR160(a-d-5p), osa-miR1320-3p, and osa-miR1425-5p were highly down regulated in PBSR. The expression of osa-miR5827 was highly abundant in Pokkali roots (PKSR and PKNR), while osa-miR1864 seemed to only be expressed in PKNR. A total of 36 miRs were down regulated in PKSR, but up regulated in PBSR (Figure 2(Ae)), and among these, osa-miR528-5p, osa-miR1850.1, and osa-miR5143a,b showed >4 fold up regulation in PBSR. 

In PK, the presence of salt caused the up regulation of 44 miRs, including osa-miR396c-3p (5-fold) and osa-miR7694-5p (3-fold) (Figure 2(Ab)), while there was down regulation of 32 miRs, including osa-miR1861a,o, osa-miR6248, osa-miR6246, and osa-miR5501, which showed >3 fold down regulation (Figure 2(Af)). The down regulation of miRs in PB like osa-miR1861c, osa-miR2863c, osa-miR444a-3p, osa-miR5339, and osa-miR5512a,b in the presence of salt was not seen in PK (Figure 2(Af)). 

To understand the specific role of miRs in root development and physiology under a saline environment, data from root tissues was compared with the data available from shoots [34]. The analysis showed that 43 miRs belonging to 27 families were specifically expressed in the root tissues. Many of these miRs have been reported to play an important role in root development [22,23,24,28,31] and salt stress [21,26,39,40,41,42,43]. Within this set, 19 miRs were abundant in PK roots and among these, seven miRs viz. osa-miR160e-3p, osa-miR1874-5p, osa-miR2106, osa-miR2120, osa-miR395o, osa-miR399k, and osa-miR6250 were expressed in the presence of salt (PKSR). 

Interestingly, it was seen that the expression levels of 65 miRs were similar (<10%) for PKNR and PBSR, indicating that the genetic machinery in Pokkali is programmed to enable the plants to adapt to salt stress. When the overall salt-induced fold dis-regulations of these miRs were compared, it was observed that the variations were less prominent (within 0-2 fold) between PKSR and PKNR. However, in PBSR/PBNR (Figure 2B), the variations were much higher, with ~40% of miRs showing >6 fold down regulation and ~40% of miRs showing >6 fold up regulation (Figure 2B). For example, osa-miR408-5p was almost 130 fold up regulated in PBSR, but was up regulated by 1 fold in PKSR (Figure 2(Aa)). 

Comprehensive analysis was performed to understand the effect of salt on miR expression patterns by comparing the fold dis-regulation patterns of PKSR and PBSR (Figure 2B; Supplementary-File 3). Color coding was used to capture the deviation in their regulation in PK roots in the presence of salt. Major variations were seen in the miRs that showed up to 4 fold up regulation in PBSR. Among the 39 miRs showing up to 2 fold up regulation in PBSR, 21 miRs were down regulated, while 17 miRs were up regulated, and 1 miR (osa-miR164c) was not expressed in PKSR. Among the 13 miRs showing 4–6 fold up regulation in PBSR, 3 miRs were down regulated, while 4 miRs were up regulated, and 6 miRs were not expressed in PKSR.

### 3.2. Validation of Salt-Regulated miRs Using the Taqman PCR Assay

**Figure 3:**
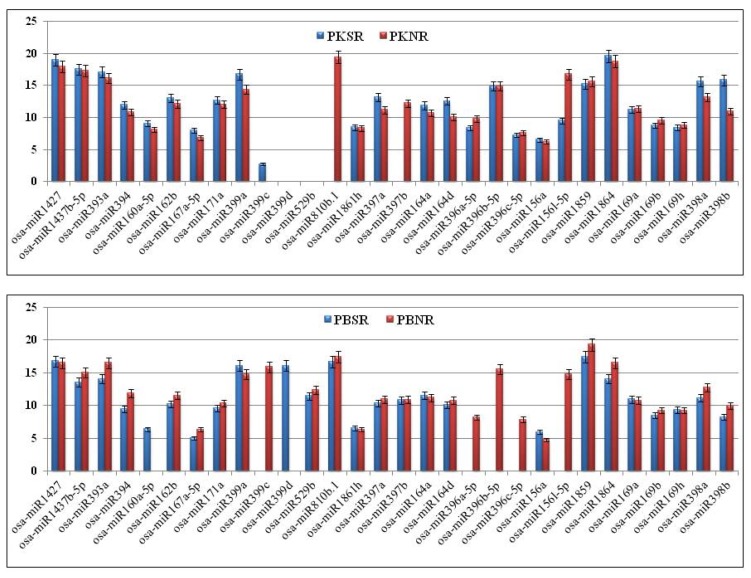
Expression profiles of osa-miRs measured by performing Taqman qRT-PCR in the root tissues of Pokkali (PK) and Pusa Basmati (PB) grown in the presence (SR) and absence (NR) of salt. All experiments were performed in triplicate and the results were normalized using 18S rRNA as the control.

To validate the expression profiles of the salt-regulated miRs in PK and PB root tissues, highly sensitive Taqman PCR assays were performed using a panel of 30 miRs (Figure 3). It was observed that the expression levels of three members of the osa-miR169 family (a, b, and h), two members of the osa-miR164 family (a and d), three members of the osa-miR396 family (a, b, and c), osa-miR393a, osa-miR394, miR1427, and miR1437-5b were similar in PKNR and PBNR. In the presence of salt, no significant change was observed in the expression of these miRs in PK, except for slight induction in osa-miR164d expression. In PBSR, the expression of osa-miR393a and osa-miR394 was down regulated, while the expression of osa-miR396 (a, b and c) could not be detected. There was no change in the expression of the other miRs.

The expression of two members of osa-miR397 (a and b) and osa-miR398 (a and b) were also similar in PKNR and PBNR. However, in the presence of salt, there was an induction in the expression of osa-miR397a and loss of expression of osa-miR397b in PK roots, while these miRs were not responsive to salt in the PB roots. Interestingly, osa-miR398 (a and b) were up regulated in PKSR and down regulated in PBSR.

The expression levels of osa-miR156a, osa-miR156l, osa-miR162b, osa-miR167a, osa-miR171a, osa-miR810b, osa-miR1861h, and osa-miR1864 were higher in PKNR than PBNR. In the presence of salt, no significant change was observed in the expression of most of these miRs in PK and PB. However, the expression of osa-miR156l and osa-miR810b was significantly reduced and absent in PKSR, respectively. In PBSR, osa-miR156l could not be detected, while there was no significant change in osa-miR810b expression. 

The expression of osa-miR160a was seen in PKNR, but not in PBNR. In the presence of salt, the miR expression was induced in PBSR, while there was a small induction in expression in PKSR. The expression level of osa-miR1859 was lower in PKNR than PBNR. No significant change was observed in its expression in PKSR, though it was down regulated in PBSR. osa-miR529b was not expressed in PK roots, whereas it was down regulated by salt in PB roots. 

The most variant expression pattern was exhibited by the osa-miR399 family. In PKNR, only osa-miR399a expression could be detected and its levels were similar to those in PBNR. This miR showed a small induction in the presence of salt in the roots of both plants. osa-miR399c was not expressed in PKNR, but showed a weak expression in PKSR, whereas it was present in PBNR, but the expression was lost in the presence of salt (PBSR). The expression of osa-miR399d could be detected in PBSR only. These results indicate that in PK roots, the miR expression patterns are genetically buffered against the effect of salt stress.

### 3.3. Target of miRs and Their Regulation in the Presence of Salt

RNAseq analysis was also performed in parallel to obtain the complete transcriptome profiles of the four libraries (Figure 1). The search for miR targets in these datasets identified 58,454 (PKNR), 48,666 (PKSR), 54,195 (PBNR), and 43,845 (PBSR) transcripts in each library (Supplementary-File 4). It was observed that under the presence of salt, there was an overall decrease in target transcripts that correlated with an increase in the number of miRs in PKSR (Figure 1). These included transcripts encoding proteinase inhibitor II protein (LOC_Os03g52360.1) that was a target of osa-miR160-3p, CHCH domain-containing protein (LOC_Os03g18420.1) which was targeted by osa-miR1869, ethylene-responsive element-binding protein (LOC_Os04g46290.1) which was targeted by osa-miR2866, and transcription factor NF-Y (LOC_Os03g48970.3, LOC_Os03g07880.3, LOC_Os03g07880.1, LOC_Os03g07880.2, LOC_Os03g48970.1, LOC_Os03g48970.2, LOC_Os03g48970.4, LOC_Os03g44540.1, and LOC_Os06g04270.1) that was a target of osa-miR169-5p. Transcripts encoding the chaperone, hsp20/alpha crystallin family protein (LOC_Os03g16020.1), and mitochondrial inner membrane translocase (LOC_Os07g41330.1) were up regulated in PKSR. These transcripts were targeted by osa-miR2120 and osa-miR319a-5p, respectively.

**Figure 4:**
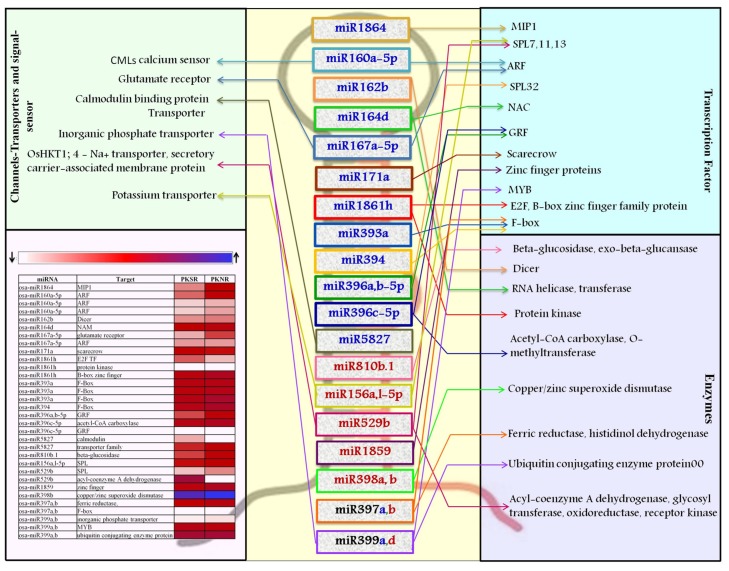
List of miRs that show stringent regulation in Pokkali root tissues grown in the presence of salt (PKSR) to modulate plant adaptability to the presence of salt. miRs up regulated in PKSR are indicated in blue. miRs down regulated in PKSR are indicated in red. The list of their predicted target transcripts indicates their functional role. Each miR:target node is depicted by its unique color code. The expression profile of targets is also shown.

The targets cleaved by the salt-regulated miRs were further validated using degradome data (Figure 1) and this identified 3649 transcripts as targets of 360 miRs (Supplementary-File 4). After filtering the degradome data through the transcriptome datasets, 182 transcripts were identified as targets of the salt dis-regulated miRs. This set included a range of transcripts coding for signal transducers, enzymes, transporters, and transcription factors. A large number of transcripts coded for proteins with unknown functions or hypothetical proteins (Supplementary-File 4, Sheet 2). The transcription factors comprised 41% of the targeted transcripts and there are prior reports on the stress association of many of them, such as miR159:MYB, miR156:SPL, miR164:NAC, miR171:GRAS, miR172:AP2-bZIP, miR319:TCP, miR393:MYB, and miR408:BCP [44,45,46,47,48,49]. The miRs showing maximum variation between PK and PB roots and their predicted targets are represented in Figure 4.

It was observed that the expression of osa-miR160a was high in PK roots and their corresponding ARF targets were down regulated, indicating that the miR160:ARF node plays an important role in root development. The expression of osa-miR162b, osa-miR164d, osa-miR167a, and osa-miR171a was also induced in PK, reflecting a concomitant change in their target transcripts to influence root development. Specific members of the miR393 and miR394 families were up regulated in PK. osa-miR393b-5p specifically targets OsFBL21 and OsFBL16, while osa-miR394 targets OsFBL32, to down regulate their expression in PKSR. 

The expression of osa-miR396 did not vary much between PKNR and PKSR, indicating a tight regulation of its targets that code for basic helix-loop-helix (bHLH) and growth regulating factor (GRF) transcription factors. During experimental validation, it was found that osa-miR529b that targets SQUAMOSA promoter-binding protein-like (SPL) transcription factors could not be detected in PK roots and was down regulated in PBSR. This indicates accumulation or up regulation of the corresponding target transcripts. The expression of osa-miR397b and osa-miR398a,b acting in the same pathway by targeting transcripts coding for ferric reductase and copper/zinc superoxide dismutase (SOD), respectively, was down regulated in PKSR. 

This analysis also identified several ion-transporters or channels and signaling molecules like kinases as targets for the miRs. osa-miR5827 was highly abundant in Pokkali roots (PKSR and PKNR) and targeted LOC_Os11g44310, encoding a putative calmodulin (CaM)-binding protein, while osa-miR812f was expressed at high levels to control CBL-interacting protein kinases (CIPK). 

**Figure 5:**
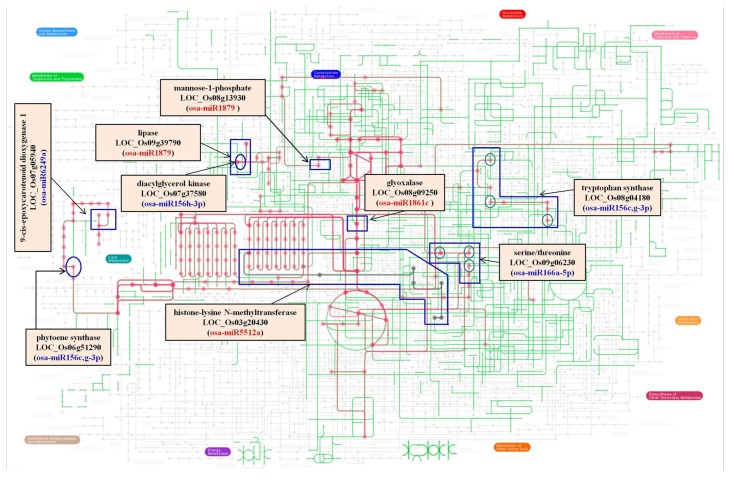
The metabolomics network employed to highlight the nodes regulated by miRs. Transcripts targeted by miRs have been marked. miRs up regulated in PKSR are indicated in blue. miRs down regulated in PKSR are indicated in red. PKSR: Pokkali root tissues grown in the presence of salt.

The transcripts identified as targets of these miRs were subjected to gene ontology analysis to understand the associated pathways (Figure 5). The results indicate that in PK roots, the overall growth and development is regulated through key transcription factors, many of which act downstream of auxin signals. The roots are also tuned to maintain ion homeostasis, regulate the ROS pathway, and maintain the energy metabolism to survive under saline environments. 

### 3.4. Mapping of Integrating miR Expression Quantitative Trait Loci (miR-eQTL)

To understand the functional implications of the miR:target nodes that are genetically activated in PK roots in the absence or presence of salt, QTL mapping was performed (Supplementary-File 5). QTL maps integrated the articulation of 53 miRs with the physiological traits involved in plant development and salt stress tolerance, such as the osmotic adjustment capacity, elongation ability, iron sensitivity, root morphology (length, weight, thickness, and number), carbohydrate content, carbon content, chlorophyll content, etc. 

**Figure 6:**
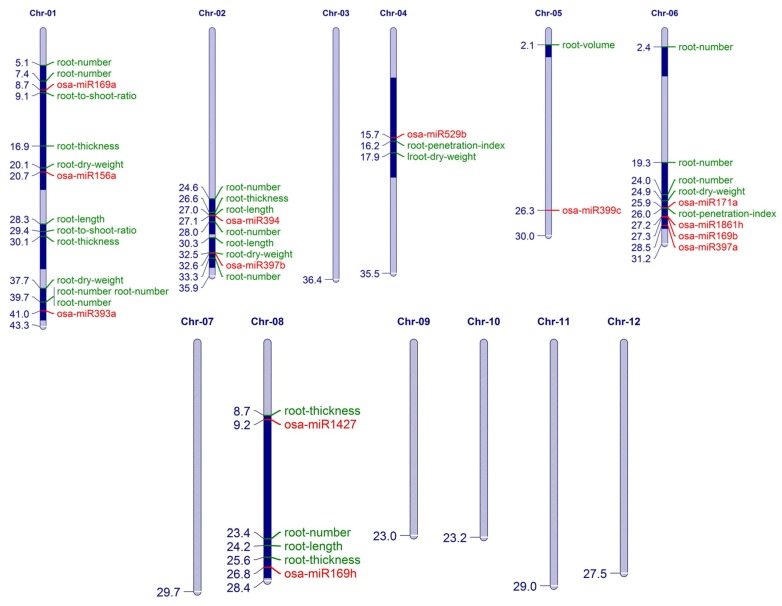
Physical map of rice chromosomes to show the association of miRs with root trait-related Quantitative trait loci (QTLs). The position of miRs and QTLs are mentioned on the left side (in Mbps) and the respective miR and QTL IDs are provided on the right side.

The miRs that mapped to QTLs related to different aspects of root growth are plotted in Figure 6. These included several miRs that were genetically abundant in PKNR, like osa-miR169(a,b,h), osa-miR171a, osa-miR1861h, osa-miR393a, osa-miR394, and miR1427. They were related to traits controlling the root number, length, thickness, dry weight, and penetration index. The miRs osa-miR156a,l, osa-miR397a,b, and osa-miR529b showed down regulation in PKSR and mapped to traits mainly related to the root dry weight. This analysis also supports the presence of an miR profile that regulates root growth under stress.

## 4. Discussion 

There are several reports on the identification of stress-regulated miRs and their involvement in cell responses to abiotic stresses, such as salinity, cold, and dehydration [40,47,50,51]. Many of the transcripts targeted by the miRs code for stress-responsive transcription factors [46,52,53,54], again indicating that miR-dependent regulation plays a crucial role in the plant stress response. In this study, the small RNA and transcriptome profiles of PK and PB root tissues grown in the presence and absence of salt were analyzed and it was observed that the miR levels in PK roots are maintained to levels that favor stress tolerance physiology of the plant. 

It has previously been proposed that miRs that are positively regulated by stress might target negative regulators of stress tolerance, while miRs that are suppressed during stress are likely to target positive regulators of stress tolerance [50]. However, there is variation in the expression of miRs across species [3], for instance, in roots of maize inbred line miR396 family members showed down regulation under salt stress at different time points, indicating the accumulation of different levels of positive regulators during the stress process, which might result in different salt sensitivities among them [26]. However, in Arabidopsis, the same miRs were found to be up regulated in response to salinity [39]. The expression of miR169, which represents one of the largest families of plant miRs, was induced after salt treatment in both Arabidopsis and rice [51].

The present study revealed that in the presence of salt, there was an increase in miR numbers by 34% in PK roots and decrease by 22% in PB roots. The negative correlation was reflected in the number of target transcripts. We have previously reported that a greater number of miRs were up regulated by salt stress in the salt-tolerant Gly transgenics (GSL/GNL) [10,55]. A comparative analysis of all four libraries indicated that the expression levels of 65 miRs were similar for PBSR and PKNR. The salt-induced variations in miR expression levels were mostly within 0–2 fold in PK roots (PKSR/PKNR), while in PB roots, ~40% of miRs showed >6 fold dis-regulation. It was calculated that among the 123 miRs showing ≥3 fold dis-regulation in PB, 86 miRs showed ≤2 fold dis-regulation in PK. Therefore, it can be readily envisaged that in the naturally salt-tolerant variety, PK, the expression of salt-regulated miRs was genetically programmed to levels that enabled the plants to grow in the presence of salt. 

**Figure 7:**
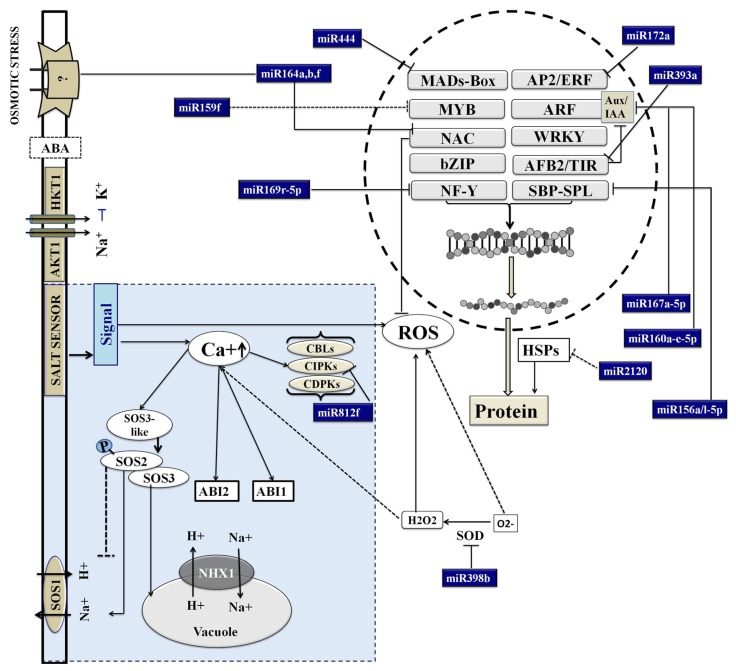
A hypothetical model built on the key Pokkali miRs and their predicted targets to represent the regulatory transcription factors. The maintenance of calcium and ROS homeostasis by miRs is also shown. These components act upstream to control the metabolic network that plays an important role in enabling plants to adapt to saline environments.

Most miRs governing root development are conserved and exert their regulation by targeting various transcription factors (Figure 7). These findings were supported by the analysis of target transcripts. For instance, PKNR had higher levels of osa-miR160a, osa-miR162b, osa-miR167a, osa-miR171a, and osa-miR1864 compared to PBNR. Individual members of miR393 and miR394 families were also up regulated in PKNR. These miRs are associated with various aspects of root development, like osa-miR160a, which regulates root tip growth, lateral root production, and gravity sensing by targeting ARF10 and ARF16 transcripts [28,41]. There are also reports on the role of auxins through ARFs in the salt stress-stimulated development of lateral root primordia [20,56,57]. In earlier reports, miR160 was shown to be down regulated under salt stress in a salt-tolerant variety of *Populus euphratica* [42] and up regulated in a salt-sensitive variety of *Vigna unguiculata* [43]. The overexpression of osa-miR160 caused a serious root cap defect in rice [28]. The osa-miR164 family is involved in the regulation of lateral root emergence and branching through its targets, the NAC transcription factors [34,58], and osa-miR167 regulates both primary and adventitious root growth under high osmotic stress by targeting ARF6, ARF8, and IAA-Ala Resistant [3,19,20]. The miR393 and miR394 families also regulate root system architecture and growth [59] and stem cell identity [60] by targeting transcripts encoding F-box proteins (including auxin receptor, TIR1), Leucine-rich repeat (LRR) proteins, and bHLH transcription factors. It was reported that the over expression of osa-miR393 in rice and Arabidopsis resulted in an increased sensitivity to salt and alkalinity stress [60]. Interestingly, miR394 was also found to be involved in the regulation of the plant response to drought and salinity stress [61]. miR394 and its target transcripts were reported to play an important role in the regulation of leaf morphological development [54]; however, their specific role in roots remains to be investigated. 

The osa-miR171 family regulates members of the scarecrow-like (SCL) transcription factor family, which play important roles in plant root and leaf development, phytohormone signaling, and stress responses [62]. It has been shown to be differentially regulated under salt stress in the roots of various plants [26]. osa-miR1864 regulates the expression of its target mRNA that encodes the ternary complex factor MIP1 leucine-zipper protein, which plays an important role in root hair development [63,64]. The osa-miR162 family regulates DCL1 [65], indicating that miR biogenesis was tightly regulated in PK roots in this study.

The low levels of miRs in PK indicate the requirement and importance of allowing the target transcription to mitigate the stress response. The expression of osa-miR1861c, osa-miR2863c, osa-miR444a-3p, osa-miR5339, osa-miR5501, osa-miR5512a,b miR6246, and osa-miR6248 was not seen or highly down regulated in PK roots. These miRs target transcripts coding for Gly I, mitochondrial 2-oxoglutarate/malate transporter, metallothionein-like protein, adaptin, homeobox (DDT domain containing) protein, Zinc finger protein, GTL1 transcription repressor, and calcium/CaM dependent protein kinase, respectively, which play important roles in responses to stress [66,67]. Similarly, a low expression of osa-miR529b and osa-miR156a,l in PK roots and PBSR favors translation of their target, the SPL transcription factor, which positively impacts root growth and the secondary accumulation of metabolites [45,48,68]. Earlier reports have suggested that osSPL2 and osSPL14 may provide tolerance to drought stress [68] and oxidative stress by regulating the expression of superoxide dismutase and peroxidase genes [69]. Similarly, a low expression of osa-miR398b in PK roots results in the accumulation of its target SOD (Figure 7). High levels of SOD help to prevent damage by oxygen free radicals [70,71].

The salt-induced variations in levels of osa-miR396c-3p and osa-miR7694-5p were tightly regulated in PKSR. They targeted the transcripts encoding for growth regulating factor (GRF) transcription factors [72] and xyloglucan galactosyltransferase KATAMARI1 [73], respectively. These proteins play important roles in controlling root growth and developmental plasticity in response to external cues [74]. A report on *Medicago truncatula* suggested that the de-regulation of miR396 affects the architecture of root growth [61]. The expression levels of osa-miR827a-c, osa-miR171f-5p, osa-miR160(a-d-5p), osa-miR1320-3p, and osa-miR1425-5p showed less dis-regulation in PKSR, but were highly down regulated in PBSR. In PKNR, these miRs are genetically maintained at steady-state low levels and their expression is not greatly influenced by salt stress, so the transcript levels are not influenced.

The other targets include transcripts coding for the SPX domain containing protein, dihydroflavonol-4-reductase (DFR), sodium-dependent vitamin C transporter, and pentatricopeptide repeat (PPR) containing protein, respectively. These transcripts play an important role in the stress tolerance response. The proteins containing the SPX domain are key players controlling a set of processes involved in Pi homeostasis [75] and inorganic phosphate (Pi) starvation has been shown to modulate responses of the root to salt stress [76]. There is also a role of the osa-miR399-regulated phosphate transporter in this pathway. Earlier reports on functional analyses of salt-responsive miR399 in the soybean root meristem indicated its crucial role in modulating root developmental plasticity [41]. DFR is an NADPH-dependent enzyme involved in the anthocyanin biosynthesis pathway. It was shown that the stress-induced up regulation of DFR affects NAD homeostasis, leading to cell-death tolerance [77]. The sodium-dependent vitamin C transporter maintains ascorbic acid homeostasis as it is essential for various metabolic and cell signaling processes. The dynamic relationship between ascorbic acid and reactive oxygen species (ROS) is well-documented [78]. The PPRs regulate the processing of chloroplast and mitochondria RNAs, stabilize mitochondrial electron transport, and reduce oxidative damage under abiotic stress [79]. 

This analysis also identified several ion-transporters or channels and signaling molecules like kinases that act to maintain cellular Ca^2+^ homeostasis and limit Na^+^ or Ca^2+^ influx at the plasma membrane [12]. This also limits the activation of Ca^2+^-induced ROS signaling (Figure 7). osa-miR5827 was highly abundant in Pokkali roots (PKSR and PKNR) and targeted LOC_Os11g44310, encoding a putative CaM-binding protein, which negatively regulates the expression of CaM to influence the intracellular Ca^2+^ concentration [17]. Similarly, osa-miR812f was expressed at high levels to control CIPK. The genetically high levels of osa-miR5827 and osa-miR812f may thus play an important role in stress responses by maintaining the expression of CaM and regulating Ca^2+^ signaling. It has been reported that under salt stress, CaM activity elevates metabolic enzymes involved in central energy pathways, which promote or at least maintain the production of energy under the limitation of photosynthesis [67].

An earlier study reported the dis-regulation of several genes and proteins in Pokkali [80]. On comparing their findings with our datasets, we could identify several transcripts in common that were targeted by 28 miRs belonging to 18 miR-families. Among these, the expression profiles of 12 transcripts showed distinct anti-correlations with the miR expression profiles, indicating their regulation by cleavage. The expression patterns of these transcripts also directly correlated with the translated proteins [80]. The analysis indicates a genetically coordinated regulation of key transcription factors, signaling molecules, ROS (Figure 7), and metabolites (Figure 5) that enables PK roots to withstand salt stress. 

## 5. Conclusions

The mapping of the rice miRNome in PK and PB roots provides new insights to understand the molecular mechanisms operative in rice cultivars showing contrasting responses to salt stress. The results obtained indicate that the adaptability of PK rice to grow in saline conditions is due to the intricate regulation of different cellular components by a variety of miRs. The expression profiles of miRs and their targets are varied in the roots of PB and PK, with controlled changes observed in PKSR and larger variations seen in PBSR. The present analysis identified a panel of miRs in PK roots that are associated with the maintenance of cellular homeostasis and development of root system architecture. QTL mapping of the root trait-associated miRs and their expression profiles have provided leads for further experimental investigations. Considering the conserved nature and spatial distribution pattern of the miRs, it can be adjudged that specific family members are involved in regulating the responses in roots. The results indicate that in PK, the miR expression levels are genetically programmed to enable the plants to withstand the salt-induced variations.

## Supplementary Materials

The following are available online at https://www.mdpi.com/2218-273X/10/4/498/s1. Supplementary-File-1: Description of library and list of accession numbers. Supplementary-File-2: Venn diagram representing the distribution of miRs and mRNA or transcripts (number within bracket) across the 4 libraries constructed from root tissues of Pokkali (PK) and Pusa Basmati (PB) grown in the presence (SR) and absence (NR) of salt. Supplementary-File-2: Venn diagram representing the distribution of miRs and mRNA or transcripts (number within bracket) across the 4 libraries constructed from root tissues of Pokkali (PK) and Pusa Basmati (PB) grown in the presence (SR) and absence (NR) of salt. Supplementary-File-3: Distribution and details of log_2_ fold salt dis-regulated known rice miRs. Expression values are represented as TPM (Transcript Per Million). Supplementary-File-4: Distribution and log_2_ fold details of target transcripts of salt dis-regulated known rice miRs. Supplementary-File-5: List of miR associated traits as determined by QTL mapping. The details of smallest and largest QTLs are provided.
