# Supplementary material for: Mapping the Salt Stress-Induced Changes in the Root miRNome in Pokkali Rice
Source: Biomolecules. 2020 Mar 25;10(4):498. doi: 10.3390/biom10040498 (PMC7226372; doi:10.3390/biom10040498)
Supplement: Supplementary file 1 [file biomolecules-10-00498-s001.zip › Supplementary-File 1.docx]

| **Accession** | **Sample_title** | **Cultivar** | **Tissue** | **Treatment** | **Assay_type** |
| --- | --- | --- | --- | --- | --- |
| SAMN14309805 | PKSR_1 | Pokkali | Root | salt stress | RNA-seq |
| SAMN14309806 | PKSR_2 | Pokkali | Root | salt stress | RNA-seq |
| GSM3039532 | PKSR | Pokkali | Root | salt stress | Small RNA |
| SAMN14309801 | PKNR_1 | Pokkali | Root | normal | RNA-seq |
| SAMN14309802 | PKNR_2 | Pokkali | Root | normal | RNA-seq |
| GSM3039531 | PKNR | Pokkali | Root | normal | Small RNA |
| SAMN14309793 | PBNR_1 | Pusa basmati | Root | normal | RNA-seq |
| SAMN14309794 | PBNR_2 | Pusa basmati | Root | normal | RNA-seq |
| GSM3039538 | PBNR | Pusa basmati | Root | normal | Small RNA |
| SAMN14309797 | PBSR_1 | Pusa basmati | Root | salt stress | RNA-seq |
| SAMN14309798 | PBSR_2 | Pusa basmati | Root | salt stress | RNA-seq |
| GSM3039537 | PBSR | Pusa basmati | Root | salt stress | Small RNA |
| SAMN14309807 | PKNR_S1 | Pokkali | Root | normal | Degradome |
| SAMN14309808 | PKSR_S2 | Pokkali | Root | salt stress | Degradome |
