# Supplementary material for: Mapping the Salt Stress-Induced Changes in the Root miRNome in Pokkali Rice
Source: Biomolecules. 2020 Mar 25;10(4):498. doi: 10.3390/biom10040498 (PMC7226372; doi:10.3390/biom10040498)
Supplement: Supplementary file 1 [file biomolecules-10-00498-s001.zip › Supplementary-File 3.pdf]

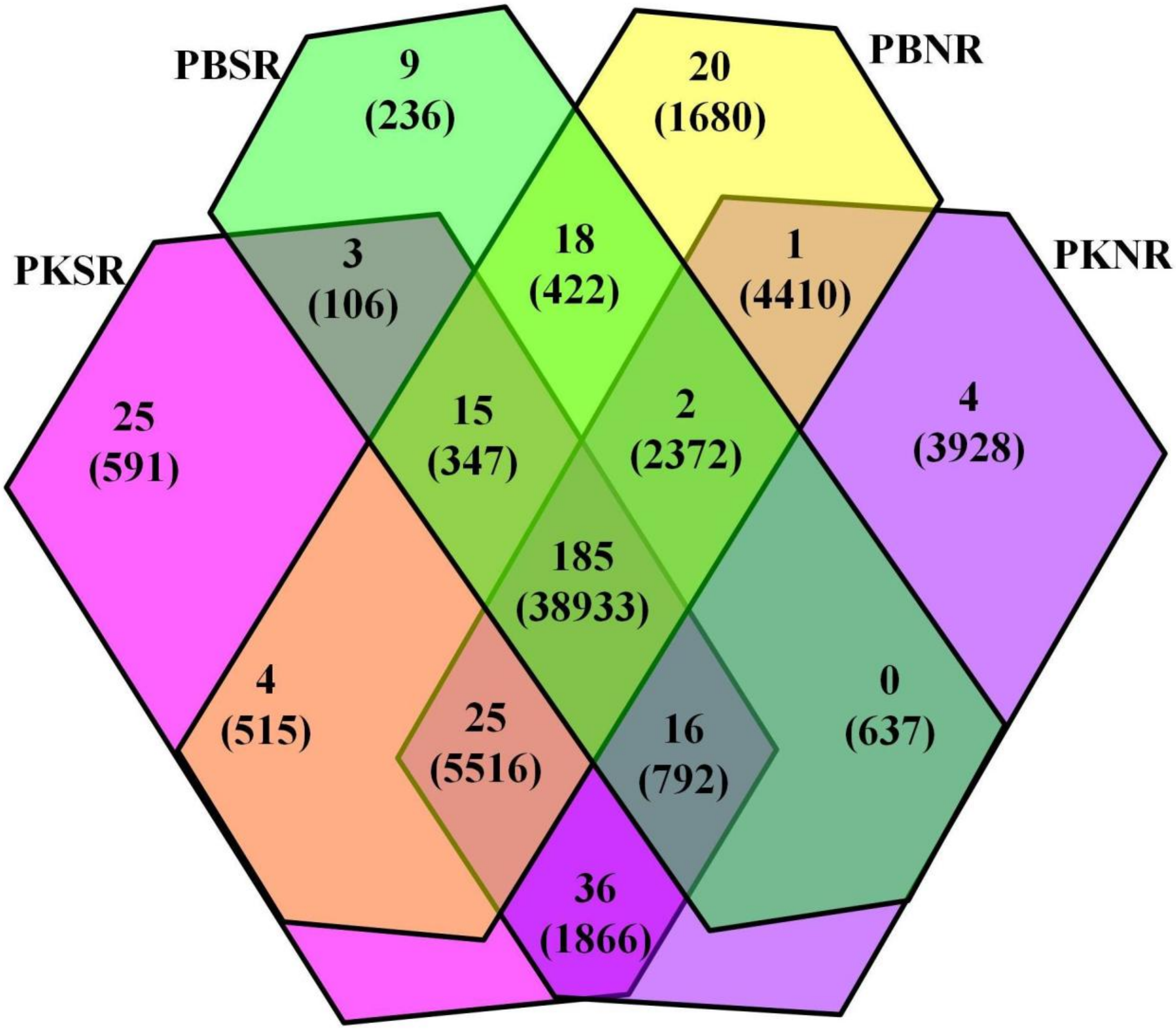

Supplementary-File 1A: Venn diagram representing the distribution of: miRs: mRNA transcript (within bracket) across the 4 root libraries under control and experimental conditions in Pusa Basmati (PBNR, PBSR) and Pokkali (PKNR, PKSR).

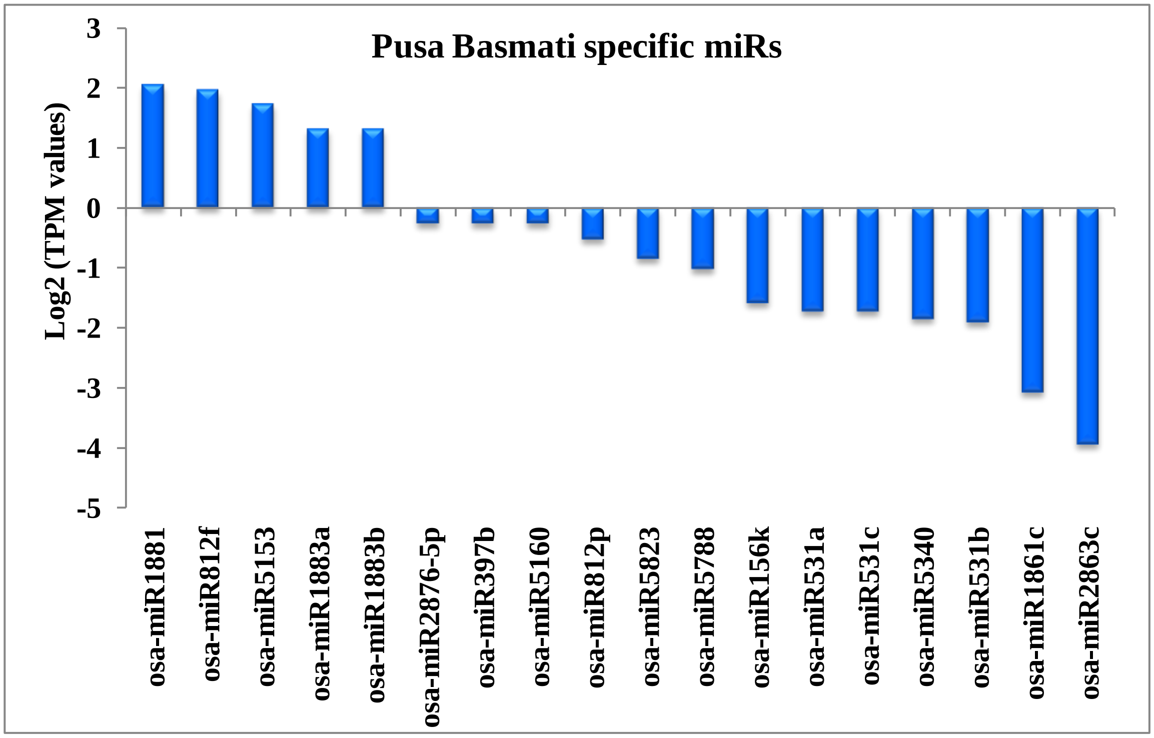

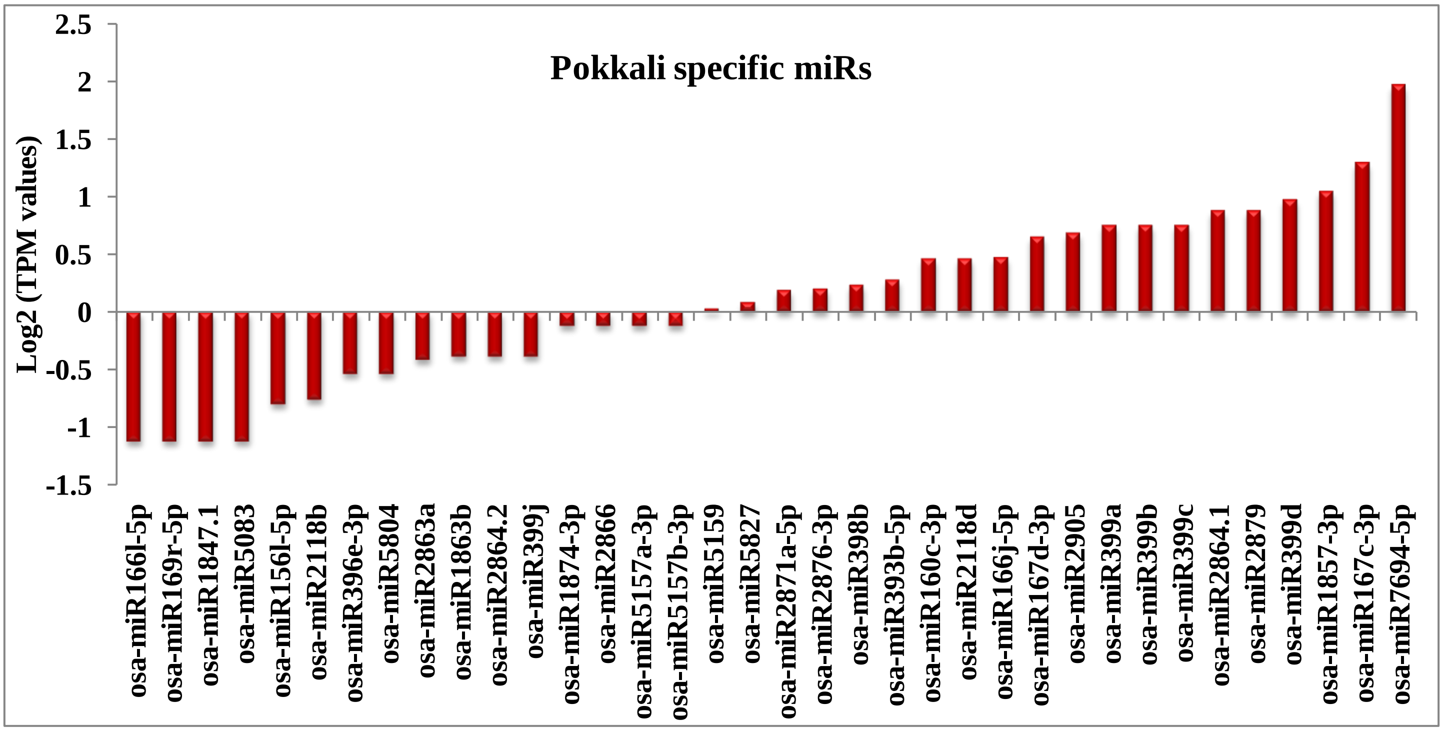

Supplementary-File 1B: Graphical representation of specific miRs: Differential miRs expression profiling of: (A) miRNAs specifically expressed miRs in PB root tissues (B) miRNAs specifically expressed miRs in PK root tissues

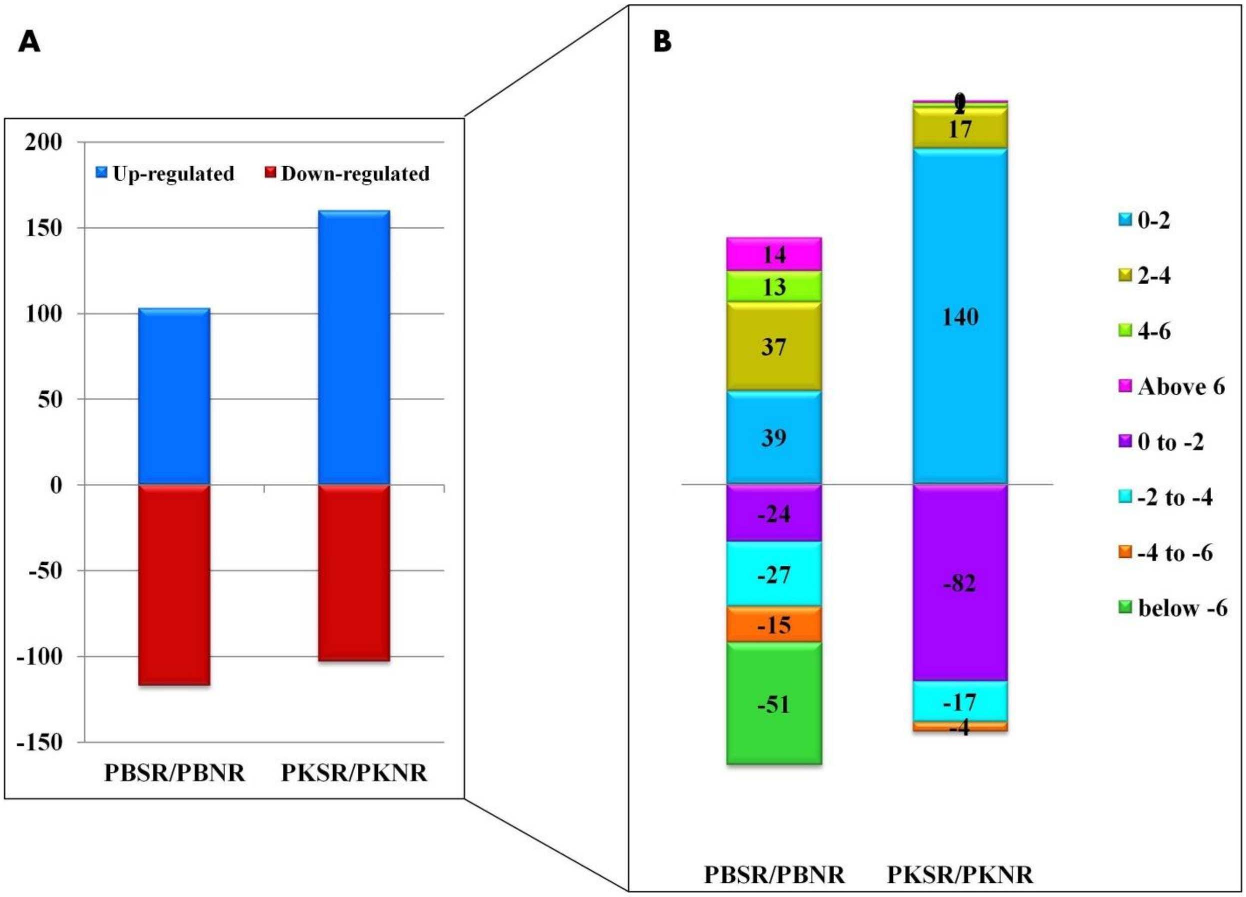

Supplementary-File 1B: **Graphical representation of the overall de-regulated** miRs: **(A)** Differential expression of miRs in PB and PK root tissues. **(B) Fold change expression of miRs** in Pusa Basmati (PBNR, PBSR) and Pokkali (PKNR, PKSR).
